# Intracluster Ion Molecule Reactions Following the Generation of Mg^+^ Within Polar Clusters

**DOI:** 10.3390/ijms12129095

**Published:** 2011-12-07

**Authors:** Edreese H. Alsharaeh

**Affiliations:** College of Science & General Studies, Alfaisal University, Riyadh 11533, Saudi Arabia; E-Mail: ealsharaeh@alfaisal.edu; Tel.: +966-50-977-1290; Fax: +966-1-215-7611

**Keywords:** solvation, gas phase clusters, time of flight

## Abstract

In this work we investigated the intracluster ion molecule reactions following the generation of Mg^+^ within the polar clusters (water, methanol, ether and acetonitrile), using time of flight mass spectrometry. In the case of Mg^+^/water and Mg^+^/methanol, dehydrogenation reactions are observed after the addition of five molecules. However, no dehydrogenation reactions are observed in the case of Mg^+^/ether or Mg^+^/acetonitrile clusters. This confirms the role of the H atom in (O–H) in the dehydrogenation reaction, and rules out any contribution from the H atom in the CH_3_ group. In addition, the magic numbers in the time of flight (TOF) mass spectra of the Mg^+^X_n_ clusters (X = H_2_O, CH_3_OH, CH_3_OCH_3_ and CH_3_CN) have been investigated. Finally, the role of ground electronic magnesium ion Mg^+^(^2^S_1/2_), and excited electronic magnesium ion Mg^+^(^2^P_1/2_) in the dehydrogenation reaction were investigated using Ion Mobility Mass spectrometry. The results offer direct evidence confirming the absence of the electronically excited, Mg^+^(^2^P_1/2_).

## 1. Introduction

Reactions in solutions are highly dependent upon the medium used, and among them ion molecule reactions represent the best systems to study solvation effects at a microscopic level [1]. Clusters offer an ideal medium to study the gradual effects of solvation on chemical reactions, which could lead to the stabilization of ionic intermediates [2].

Many groups, using different mass spectrometric techniques, have studied the solvation and the reactivity of water and methanol clusters containing group-II metal ions; magnesium, calcium, or strontium, in both the ground state and the excited electronic state [1–9]. Very intriguingly, size dependence has been found in the reaction of Mg^+^ ion with H_2_O and CH_3_OH, where the dehydrogenation reaction was observed after the addition of five water molecules to Mg^+^ [1–5].

Fuke and Iwata, *et al.* studied Mg^+^ and Ca^+^ with water, where the product MgOH^+^(H_2_O)_n − 1_ was exclusively observed [4], for 6 ≤ n < 14 in the mass spectrum. Similar product distributions were also observed for Mg^+^/D_2_O, Ca^+^/H_2_O, and Ca^+^/D_2_O systems. In these systems, ion–molecule reactions within the clusters resulted in the production of ion series, which dominates the mass spectra after certain cluster sizes. This phenomenon is known as “Product switching” [5]. Both M^+^(H_2_O)_n_ and MOH^+^(H_2_O)_n − 1_, where M^+^ = Mg^+^ or Ca^+^ were found to be formed with characteristic size distributions for the product ion switching at two critical sizes (n ~ 5 and 14). On the basis of these results, as well as the results on the successive hydration energies of MgOH^+^, the origin of the first product switching for n ~5 was ascribed to the difference in the successive hydration energies of M^+^ and MOH^+^ [7]. With increasing cluster size, the product MOH^+^(H_2_O)_n − 1_ lowers the energy of the system more than M^+^(H_2_O)_n_ product, and at n ≥ 5 the MOH^+^(H_2_O)_n − 1_ becomes the ground state of the system [4]. Castleman *et al*. [5] used flow tube instruments to examine the effects of solvation on the dehydrogenation reaction of Mg^+^(H_2_O)_n_ to produce MgOH^+^(H_2_O)_n − 1_, for n ≤ 6. The reaction was observed to occur spontaneously at room temperature for n > 4. Ligand switching reactions were used to show that Mg^+^–OH bonds are stronger than Mg^+^–H_2_O bonds. The results show that the energy required to lose an H atom decreases with the number of water molecules attached, because the magnesium ion changes its oxidation state and this results in stronger interactions with the water ligands. These experimental results differ from those of Saneketa *et al*. [3] who observed the first product switching at n = 5, and attributed this result to the low temperature of the cluster beam source. This dehydrogenation reaction was also observed by Martin Beyer *et al*. [6] using FTICR.

As for the second product switching for n~15, the origin is not self-evident. Fuke *et al*. [5] proposed two possible mechanisms: the participation of the Rydberg-type ion-pair state M^+2^(H_2_O)_n_^−^ and the formation of a new reaction product such as MOH·H_3_O^+^(H_2_O)_n − 2_. The former mechanism is based on the results for the photo-dissociation spectra of M^+^(H_2_O)_n_ and is consistent with solution processing of metal ions in bulk water. At present, the former mechanism is considered to be much more plausible for understanding the switching. However, they could not rule out the latter mechanism within the experimental data. Sanekata *et al*. also carried out molecular orbital studies to confirm the first product switching [3].

These studies raised an interesting question as to whether cluster reactions would occur in other solvent clusters (e.g., CH_3_OH), which may provide valuable information regarding the reaction mechanism. In particular, the substitution of one H in H_2_O by a CH^3^ group may help to understand the H-elimination behavior of M^+^(H_2_O)_n_. As such, the substitution of H by CH_3_ offers an opportunity to investigate the structural effects of the molecular clusters on their reactions. It would also be interesting to see whether H– elimination or CH_3_– elimination is the dominant process.

Many research laboratories have examined the interaction of Mg^+^ with methanol. An early study conducted by Uppal *et al.* [7] using an ion cyclotron resonance, ICR, spectrometer showed no reactivity of Mg^+^ ions toward methanol molecules. Kaya *et al.* [8] observed the formation of Mg^+^(CH_3_OH)_n_ (n = 1–10) in a laser ablation-molecular beam system with a magic number at n = 2. They attributed this magic number to the formation of a first solvation shell of two methanol molecules around the Mg^+^ ion. However, Woodward *et al*. [9] reported the formation of different reaction products in addition to Mg^+^(CH_3_OH)_n_ (n = 1–20) such as, Mg^+^OCH_3_(CH_3_OH)_n − 1_, (CH_3_OH)_n_H^+^, and [(CH_3_OH)_n_(H_2_O)]H^+^ (at large n values). Their experiment consisted of a supersonic expansion source for methanol clusters and a metal vapor source. The neutral Mg(CH_3_OH)_n_ clusters were ionized by electron impact. The main observation was a switching in the dominant product species at specific cluster sizes. They observed that Mg^+^(CH_3_OH)_n_ clusters were the dominant species at n < 3 while at n = 4, the dehydrogenation product, namely Mg^+^OCH_3_(CH_3_OH)_n − 1_, became dominant due to the formation of a more polarized ion core, which can be described as Mg^2+^–OCH_3_^−^. The stability of Mg^2+^–OCH_3_^−^ was attributed to the stronger interaction between Mg^2+^ and ^−^OCH_3_ and the higher enthalpy of solvation for M^+^–^−^OCH_3_ than M^+^HOCH_2_ [10].

The switching reaction between Mg^+^(CH_3_OH)_n_ and Mg^+^OCH_3_(CH_3_OH)_n − 1_ was also studied by Lu *et al.* [11] in a reflectron time of flight mass spectrometer (TOFMS) coupled with pulsed supersonic expansion source of methanol clusters and laser ablation source for Mg^+^ ions. They observed two product-switching regions. The first product switching region from Mg^+^(CH_3_OH)_n_ to Mg^+^OCH_3_(CH_3_OH)_n − 1_ at n = 5 and the second, from Mg^+^OCH_3_(CH_3_OH)_n − 1_ to Mg^+^(CH_3_OH)_n_ at n = 15. They also carried out isotope studies by substituting the H atom in OH and CH_3_ groups by D. They found that the OD group shifted the first switching region size from n = 5 to n = 6, while that of CD_3_ had no effect on the switching reaction. The isotope labeling of OH by OD also shifted the second switching from n = 15 to n = 14 while labeling CH_3_ group with CD_3_ had no effect. The authors rationalized the shift in the first switching region by suggesting that the OH bond can be broken during the hydrogen elimination and that substituting the OH with OD increases the energy required for the hydrogen elimination, thus, Mg^+^(CH_3_OH)_n_ required more methanol molecules for switching. Minor reaction products such as H^+^(CH_3_OH)_n_, Mg^+^OH(CH_3_OH)_n − 1_, Mg_2_(OCH_3_)_2_^+^(CH_3_OH)_n − 2_, and Mg_2_(OCH_3_)_3_^+^–(CH_3_OH)_n − 3_ were also observed. The latter two products indicated the formation of the dimer ion [Mg_2_^+^]. The authors explained the formation of H^+^(CH_3_OH)_n_ by electron impact ionization where the electrons were supplied from the laser plasma. The CH_3_ elimination product (MgOH^+^(CH_3_OH)_n − 1_) was not observed at smaller n and was found to be endothermic by 16 kcal/mol (for n = 1) [12–14]. The authors excluded the possibility of any contribution from electronically excited state Mg^+^(^2^P_1/2_), which is 102.2 kcal/mol [15] higher in energy than the ground state Mg^+^(^2^S_1/2_), since no MgOH^+^ was observed. *Ab initio* calculations at SCF/6-31G* level showed that three CH_3_OH molecules formed the first solvation shell around Mg^+^. While in Mg^+^OCH_3_(CH_3_OH)_4_, all the oxygen atoms were found directly bonded to the Mg^+^. This structure reflected a Mg^2+^-like core where 6 ligands formed the first solvation shell for Mg^2+^. *Ab initio* calculations also showed a ΔE of 3.237 eV for the hydrogen elimination reaction of Mg^+^(CH_3_OH). While ΔE was calculated to be 0.055 eV for hydrogen elimination of Mg^+^(CH_3_OH)_5_. The sequential binding energies for Mg^+^(CH_3_OH)_n_ [n = 1–5] were calculated to be 1.643, 1.269, 0.949, 0.756 and 0.588 eV, respectively. Those values agreed nicely with the bond dissociation energies measured by Anderson *et al.* [16] for Mg^+^(CH_3_OH)_n_ [n = 1–3] of 1.51(0.07), 1.25(0.07), and 0.95 (0.09) eV, respectively, but were lower than the value reported by Operti *et al*. [13] of 2.65 eV for Mg^+^(CH_3_OH) which was observed in a photodissociation experiment. The *ab initio* calculations on Mg^+^OCH_3_(CH_3_OH)_n − 1_ showed a more polarizable Mg^+^OCH_3_ core with charges of 1.9 and −1.3 on Mg^+^ and O of OCH_3_ respectively, compared to values of 0.9 and −0.9 on Mg^+^ and O of CH_3_OH,. The Mg^+^–O distance was shorter in Mg^+^OCH_3_(CH_3_OH)_n − 1_ than in Mg^+^(CH_3_OH)_n_. A two-cage structure ([Mg^+2^(CH_3_OH)_n_][e^−^(CH_3_OH)_m_]) was proposed for the second product switching where one cage of methanol molecules is centered around Mg^+2^ and the other cage of methanol molecules trapped the free electron which inhibited the electron transfer to methanol molecules. Then the cages were stabilized by columbic interaction. This proposed structure was based on the ion-pair structure for Mg^+^(H_2_O)_n_ [3] and Sr^+^(NH_3_)_n_ [17–19].

In this study, the solvation of Mg^+^ with polar solvents such as H_2_O, CH_3_OH, CH_3_OCH_3,_ and CH_3_CN were investigated using pulsed supersonic beam expansion coupled with laser vaporization and mass spectrometry techniques. This allows further investigation of the role of polar solvents, role of ground electronic magnesium ion Mg^+^(^2^S_1/2_), and excited electronic magnesium ion Mg^+^(^2^P_1/2_) in the dehydrogenation reaction. Such studies would show whether cluster reaction occurs in other polar solvent clusters (such as ether and acetonitrile). The results provide valuable information regarding the reaction mechanism. In particular, the substitution of one H in H_2_O by a CH_3_ group may help to understand the H-elimination behavior of M^+^(H_2_O)_n_. As such, the substitution of H by CH_3_ offers an opportunity to investigate the structural effects of the molecular clusters on their reaction. It would also be interesting to see whether H– elimination or CH_3_– elimination is the dominant process.

## 2. Results and Discussion

### 2.1. Experimental Results

#### 2.1.1. Mg^+^(H_2_O)_n_

**Figure 1:**
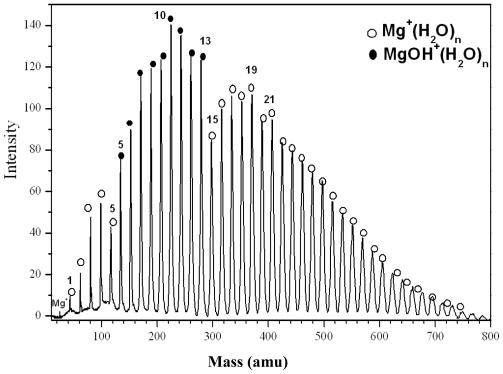
Time of flight (TOF) mass spectra of Mg^+^(H_2_O)_n_.

Figure 1 displays a typical TOF mass spectrum of water clusters containing Mg^+^. The main results can be summarized as follows: First, Mg^+^ association reactions (1) with water are observed:

(1) $${\text{Mg}}^{+}+{\text{H}}_{2}\text{O}\to {\text{Mg}}^{+}{\left({\text{H}}_{2}\text{O}\right)}_{\text{n}}$$

at n = 1–5, with a local maxima (magic number) at n= 4, observed under different experimental conditions such as nozzle width, carrier gas pressure, deflection voltage and the delay time. Second, Figure 1 also shows two switching reactions. The first switching reaction is from Mg^+^(H_2_O)_n_ to MgOH^+^(H_2_O)_n − 1_. This is observed at n ≥ 6. A second switching reaction is seen from MgOH^+^(H_2_O)_n_ to Mg^+^(H_2_O)_n + 2_ at n = 13. Interestingly, there is an intensity dip at the end of each cluster size region, where a switching is observed.

#### 2.1.2. Mg^+^(CH_3_OH)_n_

**Figure 2:**
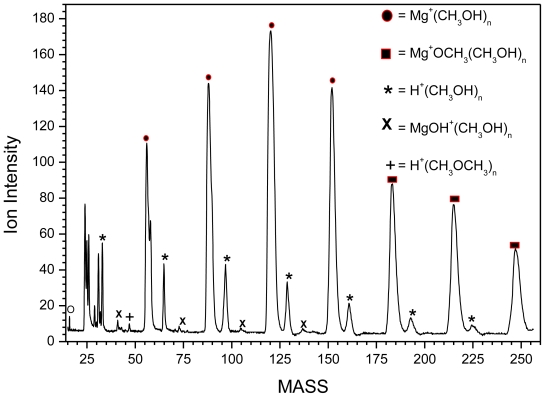
TOF mass spectra of Mg^+^(CH_3_OH)_n_.

**Figure 3:**
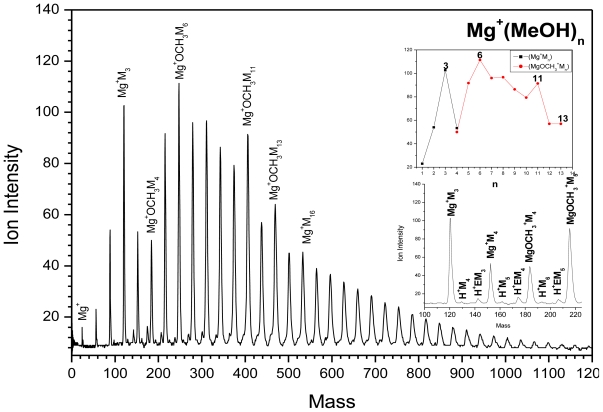
TOF mass spectra of Mg^+^(CH_3_OH)_n_. The upper inset is the ion intensity distribution as a function of cluster size, and the lower inset is TOF mass spectra from 100 amu to 250 amu.

The TOF mass spectra of methanol clusters containing Mg^+^ are displayed in Figures 2 and 3. The mass spectrum of Mg^+^(CH_3_OH)_n_ can be divided into three parts:

First, the association reactions (2) are observed

(2) $${\text{Mg}}^{+}+\text{n }{\text{CH}}_{3}\text{OH}\to {\text{Mg}}^{+}{\left({\text{CH}}_{3}\text{OH}\right)}_{\text{n}}$$

at n = 1–5, with a local maximal (magic number) at n = 3, observed under different experimental conditions such as nozzle width, pulser voltage, deflection voltage and the delay time.

Figure 3 shows that the switching reaction (3) to MgOCH_3_–(CH_3_OH)_n − 1_ is observed at n > 6. Cluster size distributions (upper inset in 2) show magic numbers at n = 7, 12 and 14.

(3) $${\text{Mg}}^{+}{\left({\text{CH}}_{3}\text{OH}\right)}_{\text{n}}\to {\text{MgOH}}^{+}{\left({\text{CH}}_{3}\text{OH}\right)}_{\text{n}-1}$$

Also Figure 3 shows a back switching reaction (4) to Mg^+^(CH_3_OH)_n_ at n ≥15.

(4) $${\text{MgOH}}^{+}{\left({\text{CH}}_{3}\text{OH}\right)}_{\text{m}}\to {\text{Mg}}^{+}{\left({\text{CH}}_{3}\text{OH}\right)}_{\text{n}}$$

Finally minor reaction products such as H^+^(CH_3_OH)_n_, Mg^+^OH(CH_3_OH)_n_, H^+^(CH_3_OCH_3_) and CH_4_^+^ are also observed as shown in Figure 3.

#### 2.1.3. Mg^+^(CH_3_OCH_3_)_n_

**Figure 4:**
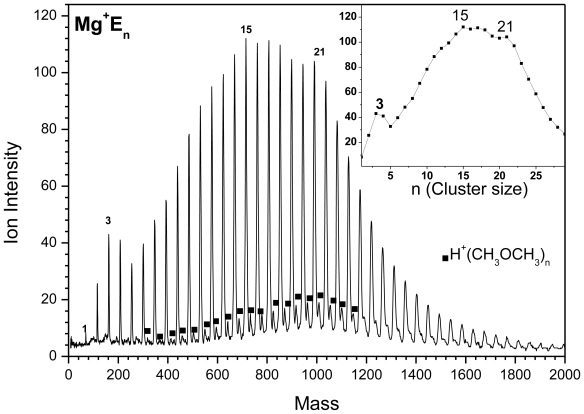
TOF mass spectra of Mg^+^(CH_3_OCH_3_)_n_, the inset is the ion intensity distribution as a function of cluster size.

The TOF mass spectrum of Mg^+^/ether clusters is shown in Figure 4. The spectrum shows the association reaction (5) according to:

(5) $${\text{Mg}}^{+}+\text{n }{\text{CH}}_{3}{\text{OCH}}_{3}\to {\text{Mg}}^{+}{\left({\text{CH}}_{3}{\text{OCH}}_{3}\right)}_{\text{n}}$$

**Figure 5:**
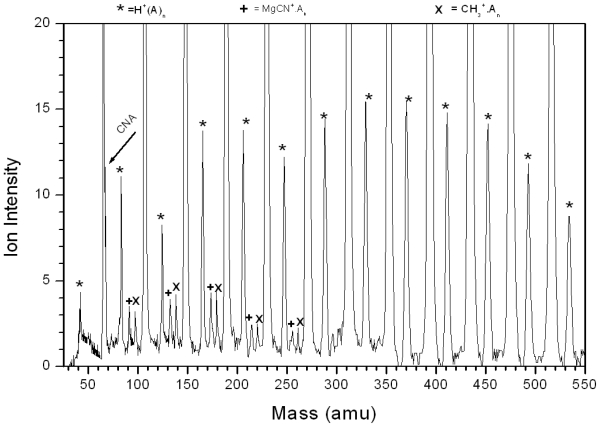
TOF mass spectra of Mg^+^(CH_3_CN)_n_.

Local maxima (magic number) at n = 3, 15 and 21 are shown in the cluster ion distribution displayed in the inset of Figure 4. These maxima are observed under different experimental conditions, such as nozzle width, pulser voltage, deflection and delay time. Figure 5 also shows protonated ether, but no dehydrogenation reaction is observed.

#### 2.1.4. Mg^+^(CH_3_CN)_n_

**Figure 6:**
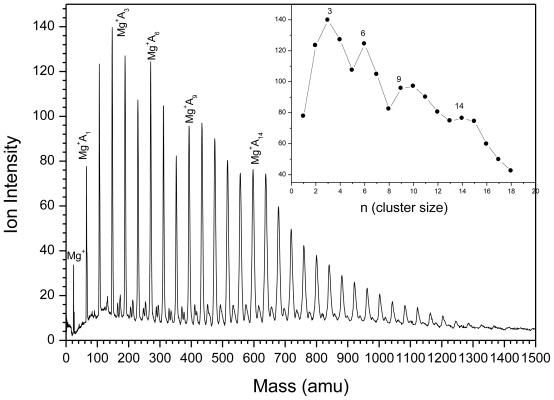
TOF mass spectra of Mg^+^(CH_3_CN)n, and the inset is the ion intensity distribution as a function of cluster size.

Figure 6 displays the TOF mass spectrum of Mg^+^(CH_3_CN)_n_ clusters system. This mass spectrum shows the association reactions (6).

(6) $${\text{Mg}}^{+}+\text{n }{\text{CH}}_{3}\text{CN}\to {\text{Mg}}^{+}{\left({\text{CH}}_{3}\text{CN}\right)}_{\text{n}}$$

Local maxima (magic numbers) at n = 3, 6, 9 and 14 are observed, as shown in the inset of Figure 6. These maxima are observed at different experimental conditions such as nozzle regime, pulser voltage, deflection and the delay time. Some other minor products are also observed as shown in Figure 6. This product ions are MgCN^+^(CH_3_CN)_n_ and CH_3_^+^(CH_3_CN)_n_ this product is a result of eliminations of CH_3_ and CN groups, respectively.

### 2.2. Discussion

Based on the results, this discussion will focus on three subjects: first, magic numbers and proposed structures; second, switching reactions; and finally, the origin of protonated clusters. The observed magic numbers in the Mg^+^X_n_ systems were X = H_2_O, CH_3_OH, CH_3_OCH_3_ and CH_3_CN as shown in Table 1.

**Figure 7:**
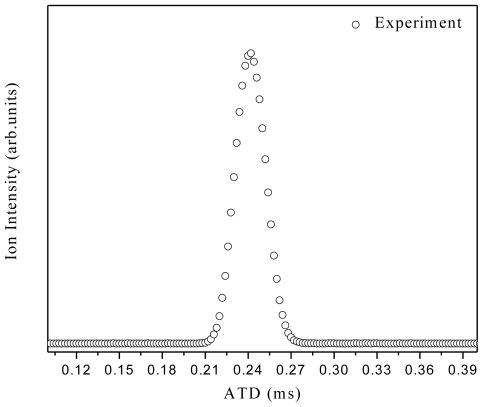
Arrival time distribution of Mg^+^ ions, produced from laser vaporization ionization LVI process. The experimental conditions are: 5 μs gate width, drift cell pressure (He) = 2.497 torr, drift cell temperature at 298.25 K and 42 V voltage difference across the cell.

In the Mg^+^/water experiments, a magic number was revealed at n = 4 for Mg^+^(water)_n_, as shown in Figure 1. This magic number may reflect the first solvation shell. However, the results from other groups suggested that the first solvation shell consists of three water molecules. The structure of Mg^+^(H_2_O)_3_ is pyramidal, with all oxygen atoms pointed directly toward the Mg^+^ ion. Comparing Mg^+^(H_2_O)_4_ with the well known magic number H^+^(H_2_O)_4_, where H_3_O^+^ is the core ion hydrogen-bonded to three water molecules, we suggest that (MgOH_2_)^+^ is the core ion in Mg^+^(H_2_O)_4_ and is shielded by three water molecules. This may explain the enhanced intensity of Mg^+^(H_2_O)_n_ series at n = 4. In the case of Mg^+^(M)_n_ where M = CH_3_OH, CH_3_OCH_3_, CH_3_CN, the results show magic numbers at n = 3. Theoretical and experimental studies [9] showed that three CH_3_OH molecules formed the first solvation shell around Mg^+^ where all the oxygen atoms were found to be directly bonded to the Mg^+^ ion. Our results indicate two switching reactions. The first being from Mg^+^(H_2_O)_n_ to MgOH^+^(H_2_O)_n − 1_ at n ≥ 5, similar to the results obtained by other groups [19]. The ion product corresponds to stabilized Mg^2+^. This conclusion was based on an energy argument which showed that the production of MgOH^+^(H_2_O)_n_ is more favorable at n ≥ 5. On the other hand, Mg^+^(H_2_O)_n_ is energetically more favorable at n < 5. This is due to the fact that the endothermic reaction (Mg^+^(H_2_O)_1_ → MgOH^+^ + H) requires ~3.3 eV [5]. Adding five water molecules compensates for this endothermicity. Thus, the ion product MgOH^+^ becomes dominant in the mass spectrum at n ≥ 5. All the experimental results are consistent with theoretical calculations which show it is energetically favorable to form Mg^2+^ and OH^−^ at a higher degree of salvation [20]. Comparing Mg^+^/water clusters to Mg^+^/methanol clusters, they appear to behave in the same manner in terms of switching reactions. This may lead us to the conclusion that only one H atom is involved in the dehydrogenation reaction. On the other hand, no dehydrogenation reaction was observed in the case of ether or acetonitrile clusters. This may confirm the role of H (in (O–H)) atom in the dehydrogenation reaction. The H atom from the CH_3_ group offers no contribution even though it is energetically more favorable. From the Mg^+^/methanol experiment, minor reaction products such as H^+^(CH_3_OH)_n_, Mg^+^OH(CH_3_OH)_n_, H^+^(CH_3_OCH_3_) and CH_4_^+^ were observed. These series were also observed by Lu *et al*. [11]. The authors explained the formation of H^+^(CH_3_OH)_n_ by electron impact ionization with the electrons supplied from the laser plasma. The CH_3_ elimination product (MgOH^+^(CH_3_OH)_n_) was not observed at smaller n, and was found to be endothermic by 118 kcal/mol (for n = 1) [12,13]. The authors excluded any contribution from electronically excited state Mg^+^ (^2^P_1/2_), since no MgOH^+^ was observed, which is 102.2 kcal/mol [15] higher in energy than the ground state Mg^+^ (^2^S_1/2_). However, our results showed the formation of MgOH^+^ as shown in Figure 2. To investigate whether there is any contribution from the electronically excited state of Mg^+^ (^2^P_1/2_), we used VCU mass-selected ion mobility system [21–23] to separate ground and electronically excited states of Mg^+^. Our results showed only one peak in the arrival time distributions, as shown in Figure 7. This peak was assigned to the ground state Mg^+^ (^2^S_1/2_). This is direct evidence confirming the absence of the electronically excited Mg^+^(^2^P_1/2_), which may lead us to a conclusion that the Electron Impact process is responsible for the formation of the minor product ions that are observed in the mass spectra.

## 3. Experimental Section

**Figure 8:**
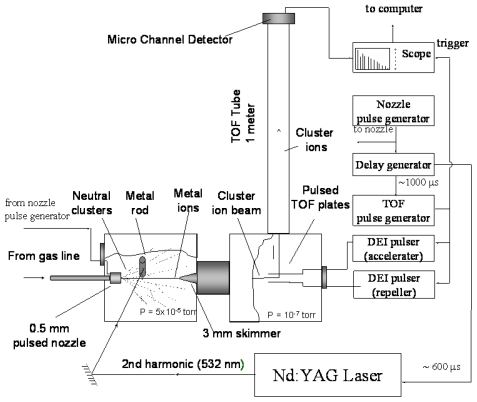
Experimental setup of time of flight mass spectrometer (TOFMS) for the generation of metal cation, containing molecular clusters.

The experiments were performed using a home built Wiley-McLaren [24] type time of flight mass spectrometer (TOFMS), as shown in Figure 8. The set up consists of two vacuum chambers. The source chamber is pumped by a Varian VHS-6” diffusion pump, 3000 L/s (He). This chamber houses a metal target, which is mounted on a sample-holder placed 8 mm downstream from a pulsed valve/nozzle. The pulsed valve/nozzle, a General Valve series 9, coupled with a 200-μm diameter conical nozzle is used to generate the cluster beam by supersonic expansion of a gas mixture of the reagent vapor, seeded in He (ultra high purity Spectra Gases 99.999%) with a backing pressure of 3–8 atm, through a 0.5 mm nozzle. The molecular beam containing cluster ions skimmed through a 5 mm diameter skimmer cone. This skimmer separates the first chamber and second chamber. The second chamber, the flight tube chamber, contains the extraction region, which consists of three plates arranged in the order repeller, accelerator and ground plate. There are also two deflection plates located perpendicular to these three plates. The flight tube chamber is differentially pumped by a Varian VHS-6” diffusion pump, 1500 L/s (He). The operating pressure is 5 × 10^−5^ torr for the source chamber and 1 × 10^−7^ torr for the flight tube chamber. A typical experiment starts with the opening of the pulsed valve, followed by the generation of the metal cations by vaporization of a metal target inside the source chamber, using a second harmonic Nd:YAG laser at ~10 mJ/pulse, with a repetition rate of 5–8 Hz. The laser-ablated metal ions perpendicularly across the expansion stream 8 mm from the ablation target where they react with the neutral molecular clusters. The result is an ionic cluster beam, collimated with a 5 mm diameter skimmer 10 cm down the stream, which then travels to the second high vacuum chamber, differentially pumped, passing symmetrically between the repeller and the accelerator. This chamber is maintained at an operating pressure of 1.0 × 10^−7^ torr. The cationic clusters are then introduced into the extraction region, which consists of three pulsed grids: repeller, accelerator and ground plates. The accelerator and the ground plates are constructed from 90% transmission copper wire mesh. The cluster ion beam is then analyzed using a pulsed TOF mass spectrometer. Most of the cluster ions were studied using a one-Pulser setup. One Pulser setup consists of DEI GRX-3.0K-H pulsed high voltage divider. The dividing ratio is 8/10 for accelerator and repeller respectively. After acceleration, the product cations travel along the field-free 1 m flight tube, and are then detected by a microchannel-plate detector (MCP) located at the end of the flight tube. The current from the detector is then amplified and recorded by a Tektronix TDS 210 oscilloscope as a function of time. It is then converted into mass using a linear square fit. The mass spectra are usually accumulated for 128 laser shots and are transferred to the computer using a National Instrument interface board model (GPIB-PCII). The delay times between the nozzle opening, the laser firing, and the ion extraction are adjusted to obtain the maximum signal intensities of the desired range of cluster size distribution.

## 4. Conclusions

In this work we present evidence of the role of the H atom in (O–H) in the dehydrogenation reaction and rule out any contribution from the H atom in the CH_3_ group. Also, the role of ground electronic magnesium ion Mg^+^(^2^S_1/2_), and excited electronic magnesium ion Mg^+^(^2^P_1/2_) in the dehydrogenation reaction were investigated. The results offer direct evidence confirming the absence of the electronically excited Mg^+^(^2^P_1/2_).

## Figures and Tables

**Table 1:** Summary of the magic numbers obtained from time of flight mass spectrometer (TOFMS).

Solvated Cluster ions	Magic Numbers			
Mg^+^(H_2_O)_n_	4	17	19	21
MgOH^+^(H_2_O)_n_	5	10	13	-
Mg^+^(CH_3_OH)_n_	3	16	-	-
MgOCH_3_^+^(CH_3_OH)_n_	6	11	13	-
Mg^+^(CH_3_OCH_3_)_n_	3	15	21	-
Mg^+^(CH_3_CN)_n_	3	6	9	14
